# Targeting Nuclear NOTCH2 by Gliotoxin Recovers a Tumor-Suppressor NOTCH3 Activity in CLL

**DOI:** 10.3390/cells9061484

**Published:** 2020-06-18

**Authors:** Rainer Hubmann, Susanne Schnabl, Mohammad Araghi, Christian Schmidl, André F. Rendeiro, Martin Hilgarth, Dita Demirtas, Farghaly Ali, Philipp B. Staber, Peter Valent, Christoph Zielinski, Ulrich Jäger, Medhat Shehata

**Affiliations:** 1 Department of Internal Medicine I, Division of Hematology & Hemostaseology, Medical University of Vienna, 1090 Vienna, Austria; susanne.schnabl@meduniwien.ac.at (S.S.); mohammad.araghi@meduniwien.ac.at (M.A.); martin.hilgarth@meduniwien.ac.at (M.H.); dita.demirtas@meduniwien.ac.at (D.D.); alifarghly.1980@gmail.com (F.A.); philipp.staber@meduniwien.ac.at (P.B.S.); peter.valent@meduniwien.ac.at (P.V.); christoph.zielinski@meduniwien.ac.at (C.Z.); ulrich.jaeger@meduniwien.ac.at (U.J.); 2 Comprehensive Cancer Center Vienna, Drug and Target Screening Platform DTS, Medical University of Vienna, 1090 Vienna, Austria; 3 CeMM Research Center for Molecular Medicine of the Austrian Academy of Sciences, 1090 Vienna, Austria; christian.schmidl@ukr.de (C.S.); arendeiro@cemm.oeaw.ac.at (A.F.R.); 4 Regensburg Center for Interventional Immunology (RCI), Franz-Josef-Strauss-Allee 11, 93053 Regensburg, Germany; 5 Ludwig Boltzmann Institute for Hematology and Oncology, Medical University of Vienna, 1090 Vienna, Austria

**Keywords:** chronic lymphocytic leukemia (CLL), *NOTCH2*, *NOTCH3*, *FCER2* (CD23), *NR4A1*, gliotoxin, RO4929097, γ-secretase inhibitors, ATAC-seq, binary cell fate decision, positive/negative selection, CD5+ B-cell homeostasis

## Abstract

NOTCH signaling represents a promising therapeutic target in chronic lymphocytic leukemia (CLL). We compared the anti-neoplastic effects of the nuclear NOTCH2 inhibitor gliotoxin and the pan-NOTCH γ-secretase inhibitor RO4929097 in primary CLL cells with special emphasis on the individual roles of the different NOTCH receptors. Gliotoxin rapidly induced apoptosis in all CLL cases tested, whereas RO4929097 exerted a variable and delayed effect on CLL cell viability. Gliotoxin-induced apoptosis was associated with inhibition of the *NOTCH2/FCER2* (CD23) axis together with concomitant upregulation of the *NOTCH3/NR4A1* axis. In contrast, RO4929097 downregulated the *NOTCH3/NR4A1* axis and counteracted the spontaneous and gliotoxin-induced apoptosis. On the cell surface, NOTCH3 and CD23 expression were mutually exclusive, suggesting that downregulation of NOTCH2 signaling is a prerequisite for NOTCH3 expression in CLL cells. ATAC-seq confirmed that gliotoxin targeted the canonical NOTCH signaling, as indicated by the loss of chromatin accessibility at the potential NOTCH/CSL site containing the gene regulatory elements. This was accompanied by a gain in accessibility at the NR4A1, NFκB, and ATF3 motifs close to the genes involved in B-cell activation, differentiation, and apoptosis. In summary, these data show that gliotoxin recovers a non-canonical tumor-suppressing NOTCH3 activity, indicating that nuclear NOTCH2 inhibitors might be beneficial compared to pan-NOTCH inhibitors in the treatment of CLL.

## 1. Introduction

Chronic lymphocytic leukemia (CLL) is considered an antigen-driven B-cell neoplasm, characterized by clonal expansion of mature CD5+ B-lymphocytes [1,2,3,4,5,6]. Despite its clinical heterogeneity, a consistent feature of CLL cells is the overexpression of NOTCH1 and NOTCH2 [7,8,9,10]. *NOTCH1* is affected by gain-of-function mutations in a subset of CLL cases (10 to 15%), where it is considered to be an independent prognostic marker associated with disease progression [11,12,13,14,15,16,17]. The high nuclear NOTCH2 activity is not only a hallmark of all CLL cases—where it is associated with the expression of the B-cell activation/differentiation marker CD23—but is also functionally linked with CLL cell viability [7,8,18].

The conserved *NOTCH* gene family (*NOTCH1-4*) encodes transmembrane receptors that regulate a wide variety of differentiation processes by modulating binary cell fate decisions in response to external signals [19,20,21,22]. Canonical NOTCH signaling is induced by ligand binding, the intracellular domain of NOTCH (N^IC^) is released by a series of proteolytic events involving γ-secretase followed by translocation to the nucleus, where it acts as context and cell type specific transcription factor on CSL (for CBF1, Suppressor of Hairless, and LAG-1)-responsive genes like *FCER2* (CD23) in CLL cells [7,18,20,21,22]. However, non-canonical NOTCH signaling also exists and involves the activation of NFκB [23]. In the murine system, *Notch2* is implicated in the development of marginal zone (MZ) B2 B-cells and of Cd5+ (B-1a) B-lymphocytes [24], and is indispensable for CLL initiation in Cd5+ (B-1a) B-cells [25].

Deregulation of NOTCH signaling is observed in an increasing number of human neoplasms, where the individual NOTCH receptors act either as oncogenes or as tumor suppressors, depending on the cellular context and microenvironment [20,26,27]. Therefore, targeting oncogenic NOTCH, for example with γ-secretase inhibitors (GSI), represents a promising therapeutic strategy in the treatment of NOTCH-associated tumors/leukemias [27,28,29,30,31]. In a first attempt to address this issue, we found that the majority of CLL cases express GSI-resistant NOTCH2/CSL transcription factor complexes and did not respond to the selective GSI DAPT [18]. In contrast, targeting nuclear NOTCH2 with the *Aspergillum*-derived NOTCH2/CSL transactivation inhibitor, gliotoxin efficiently induced apoptosis in CLL cells by a mechanism involving the induction of the *NOTCH3* and the *NR4A1* gene on the mRNA level [32]. However, the global effect of gliotoxin on the complex and interconnected signal transduction pathways and the role of NOTCH3 in CLL cells remains to be determined.

In the current study, we extended our prior work and compared the anti-neoplastic effects of gliotoxin and the GSI RO4929097 [29,31,33] in a reasonable cohort of well-characterized CLL cases. Here we show that the inhibition of NOTCH2 signaling by gliotoxin is associated with the recovery of a potentially non-canonical tumor suppressing NOTCH3 activity in CLL cells. Furthermore, assays for transposase-accessible chromatin with high-throughput sequencing (ATAC-seq) revealed that gliotoxin treatment is associated with prominent changes in the epigenetic landscape in CLL cells.

## 2. Materials and Methods

### 2.1. Patients’ Characteristics and Sample Collection

Heparinized peripheral blood was obtained from 33 CLL patients after signed informed consent (MUW-IRB approval numbers 495/2003, 11/2005, and 36/2007). Peripheral blood mononuclear cells (PBMC) were isolated using Ficoll-Hypaque (GE Healthcare, Uppsala, Sweden) centrifugation. CLL cases were screened for characteristic CLL chromosomal aberrations by FISH analysis. The *IGHV* and *NOTCH1* mutational status was determined by Sanger sequencing (LGC Genomics, Berlin, DE). The GSI sensitivity of nuclear NOTCH2 was determined by quantification of DNA-bound NOTCH2/CSL transcription factor complexes in CLL cells ±0.5 µM RO4929097 after one day of incubation using electrophoretic mobility shift assays (EMSA), essentially as described [18]. The NOTCH2 (C651.6DbHN) antibody used for the supershift/interference assays was obtained from the Developmental Studies Hybridoma Bank (University of Iowa, Department of Biological Science, Iowa City, IA, United States). The patients’ characteristics are summarized in Table 1.

### 2.2. Chemical Reagents, Compounds, and Culture

RO4929097 was purchased from Selleckchem (Houston, TX, USA). DAPT (N-[N-(3,5-Difluorophenacetyl)-L-alanyl]-S-phenylglycine t-butyl ester); gliotoxin, the NFκB activation inhibitor 6-amino-4-(4-phenoxyphenylethylamino)quinazoline, and PMA (Phorbol-12-myristat-13-acetat) were obtained from Merck Millipore (Darmstadt, DE). All compounds were reconstituted in dimethyl sulfoxide (DMSO). PBMCs from CLL patients were cultured in RPMI 1640 supplemented with 10% heat-inactivated fetal calf serum (FCS), 2 mM Glutamine, 100 U/mL penicillin, and 100 mg/mL streptomycin (all reagents were obtained from Gibco, Life Technologies Inc., Paisley, UK). 

### 2.3. Flow Cytometry and Detection of Cell Viability

Antibodies against CD5, CD19, and CD23 were purchased from eBioscience (San Diego, CA, USA). The anti-human NOTCH3 antibody (Clone MHN3-21) was purchased from BioLegend (San Diego, CA, USA). Flow cytometry was performed on a FACSCalibur^TM^ using CellQuest Pro software (Becton Dickinson, San Jose, CA, USA). AnnexinV and propidium iodide staining was performed to estimate the percentages of cells undergoing apoptosis. Apoptosis was calculated as the sum of early apoptotic (Ax+/PI−) and late apoptotic/necrotic (Ax+/PI+) cells using a kit from eBioscience (San Diego, CA, USA). Cell viability/metabolic activity was evaluated by a nonisotopic MTT (3-(4,5-dimethylthiazolyl-2)-2,5-diphenyltetrazolium bromide) assay (Ez4U) (Biomedica, AT).

### 2.4. Reverse Transcription Polymerase Chain Reaction (RT-PCR) Analysis

Total RNA was extracted using the TRI Reagent^®^ isolation system (Sigma-Aldrich, St Louis, MO, USA). M-MLV reverse transcriptase and GoTaq PCR kits (Promega, WI, USA) were used for semi quantitative RT-PCR. The *MYC* primer sequences used in this study read as follows: forward 5’-GAAAACAATGAAAAGGCCCC-3’ and reverse 5’-TTCCTTACGCACAAGAGTTC-3’. Primer sets for *NOTCH1*, *NOTCH2*, *NOTCH3*, *FCER2*, *NR4A1*, and *ACTB* were published elsewhere [32]. PCR bands were stained with GelRedTM (Biotium, Fremont, CA, USA) and visualized using the ChemiDocTM gel imaging system from Bio-Rad (Hercules, CA, USA).

### 2.5. Gene Silencing by RNA-Interference

The siRNA duplexes (siRNAs) for *NOTCH3* (ON-TARGETplusTM) and the controls (RISC-free Co-siRNA, and siGLO red transfection indicator) were obtained from Dharmacon (Lafayette, CO, USA). Transfection of siRNAs into the CLL cells was performed by using the lipid reagent siLentFect^TM^ from Bio-Rad Laboratories (Hercules, CA, USA). The transfection efficiency was determined by FACS and varied from 70 to 90%.

### 2.6. ATAC-Seq

Accessible-chromatin mapping on the CLL cells was performed using the ATAC-seq method with minor modifications together with the ATAC-seq processing pipeline, as described previously [34,35]. Principle component analysis (PCA) was performed on the quantile-normalized log-transformed values of chromatin accessibility across all the accessible sites discovered in all samples, and DESeq2 was used to detect the differential regions between treatment timepoints across patients [36]. Regions with an FDR-adjusted p-value smaller than 0.05 were selected and clustered using the Euclidean distance and complete linkage, from which two clusters representing the earliest branching point were extracted. HOMER [37] was used for de novo motif finding on the region clusters and LOLA [38] for enrichment in previously existing location-based datasets. Genes assigned to the two clusters of regions were enriched using the Enrichr tool [39].

## 3. Results

### 3.1. Dose and Time-Dependent Effects of Gliotoxin and GSI on CLL Cell Viability

Eighteen CLL cases (Table 1) were subjected to MTT assays and the dose-dependent effect of gliotoxin, RO4929097, and DAPT (0.01 to 10µM) on cell viability was determined after 3 and 7 days of incubation. 

**Figure 1:**
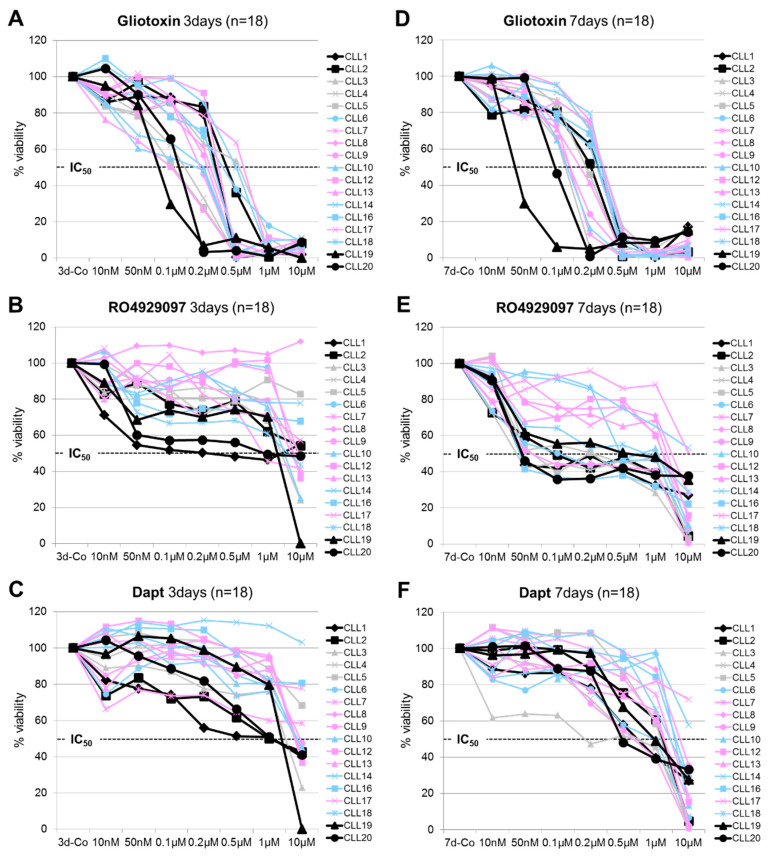
The dose- and time-dependent effect of gliotoxin, RO4929097, and DAPT on CLL cell viability in vitro. Eighteen CLL cases, including 4 cases with the recurrent *NOTCH1ΔCT* mutation (black lines), 6 cases expressing GSI-R nuclear NOTCH2 (pink lines), and 5 cases expressing GSI-S nuclear NOTCH2 (blue lines), were subjected to MTT assays and the relative inhibition of cell viability in response to the indicated drug concentrations was determined after 3 (**A**–**C**) and 7 days (**D**–**F**), respectively. The percent inhibition relative to the controls was calculated from the mean OD (optical density) values from CLL samples cultured in triplicates. The IC_50_ of the individual compounds is indicated.

After 3 days, gliotoxin efficiently decreased CLL cell viability (IC50 between 0.1 µM and 1 µM), whereas RO2949097 and DAPT were only partly effective in a subset of patient samples, including those cases characterized by the *NOTCH1ΔCT* mutation (Figure 1A–C). The mean inhibition of CLL cell viability (±SD) in the *NOTCH1* wild type versus *NOTCH1ΔCT* mutated CLL cases at an inhibitor concentration of 0.5 µM after 3 days was as follows: 84% (±23%) versus 87% (±16%) for gliotoxin, 14% (±13%) versus 36% (±14%) for RO4929097, and 10% (±14%) versus 33% (±16%) for DAPT, respectively. 

After 7 days, the response to RO4929097 was more prominent and 11 out of the 18 treated CLL cases (61%) reached IC_50_ levels at a drug concentration of 0.5 µM (Figure 1E) and, thus, were phenotypically considered as GSI sensitive. The *NOTCH1ΔCT* mutated CLL cases and/or CLL cases expressing the GSI-sensitive (GSI-S) nuclear NOTCH2 clustered in this GSI-sensitive group. In contrast, CLL cases expressing GSI-resistant (GSI-R) nuclear NOTCH2 and with the wild type (wt) *NOTCH1* status clustered in the GSI-resistant group. Maximal responses were already achieved at 0.1 µM RO4929097 and increasing amounts did not significantly enhance its effect on the CLL cells. The drop in CLL cell viability at higher drug concentrations (10 µM) after this plateau phase might be attributed to off-target effects (Figure 1B,E) [40]. The mean inhibition of CLL cell viability (±SD) in the *NOTCH1wT* versus *NOTCH1ΔCT* mutated CLL cases at an inhibitor concentration of 0.5 µM after 7 days was as follows: 95% (±6%) versus 92% (±5%) for gliotoxin, 40% (±20%) versus 54% (±7%) for RO4929097, and 22% (±17%) versus 38% (±12%) for DAPT, respectively.

### 3.2. GSI Inhibited Spontaneous Apoptosis in Early Clinical Stage-Derived CLL Samples Expressing GSI Resistant Nuclear NOTCH2

The observed delayed effect of GSI on CLL cell viability in the MTT assays suggests that GSI primarily affect the metabolic activity rather than directly inducing apoptosis. Therefore, we measured the percentage of apoptotic CLL cells (*n* = 16) after exposure to equal doses (0.5 µM) of gliotoxin, RO4929097, and DAPT by AnnexinV/PI staining.

**Figure 2:**
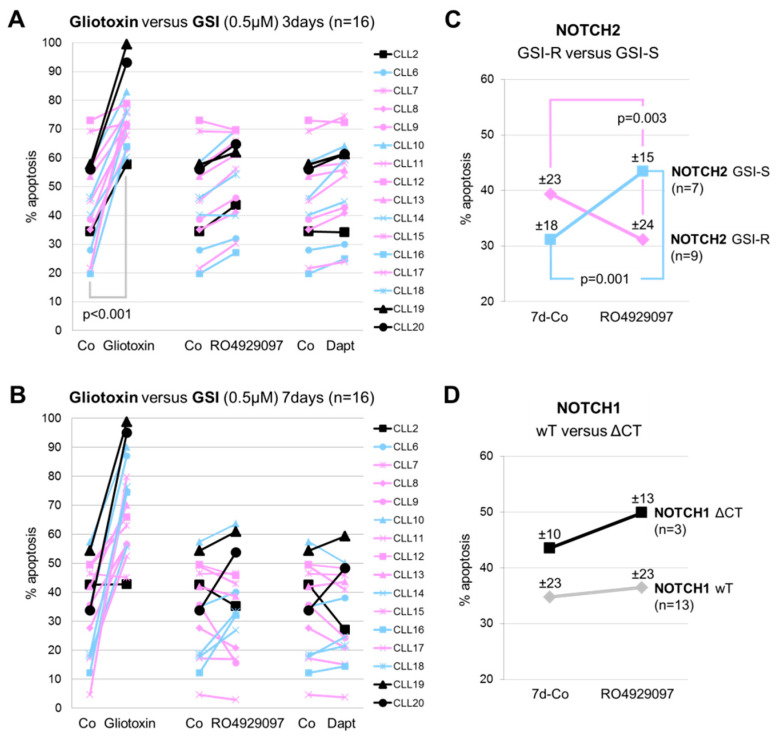
Effect of gliotoxin, RO4929097, and DAPT on apoptosis in CLL cells. Sixteen CLL cases, including 3 cases with the recurrent *NOTCH1ΔCT* mutation (black lines), 8 cases expressing GSI-R nuclear NOTCH2 (pink lines), and 5 cases expressing GSI-S nuclear NOTCH2 (blue lines), were subjected to FACS analysis and the induction/inhibition of apoptosis in response to the indicated drug concentrations relative to controls was determined after 3 (**A**) and 7 days (**B**) in suspension cultures. The mean effects (±SD) of 0.5 µM RO4929097 on the percentage of apoptotic CLL cells after 7 days in relation to the GSI sensitivity of NOTCH2 (**C**) and the *NOTCH1* mutational status (**D**) are indicated.

After 3 days, gliotoxin significantly induced apoptosis in all CLL samples (*p* < 0.001), whereas the effects of GSI were moderate (Figure 2A). After 7 days, GSI had a variable effect on CLL cell apoptosis (Figure 2B). RO4929097 increased apoptosis in CLL cases expressing GSI-S nuclear NOTCH2 (*p* = 0.003; *n* = 7) and, surprisingly, decreased apoptosis in CLL cases expressing GSI-R nuclear NOTCH2 (*p* = 0.001; *n* = 9) (Figure 2C), irrespective of the *NOTCH1* mutational status (Figure 2D). Interestingly, the anti-apoptotic effect of GSI was restricted to CLL samples derived from Rai/Binet I/A and II/B patients (CLL2, CLL7, CLL8, CLL9, CLL11, CLL12, CLL13, CLL15, and CLL17), whereas a pro-apoptotic effect of GSI was mainly observed in CLL samples derived from Rai/Binet IV/C patients (5 out of 7; CLL6, CLL10, CLL14, CLL16, and CLL20).

### 3.3. The Inhibition of Spontaneous Apoptosis by RO4929097 is Associated with Inhibition of Recovered NOTCH3 mRNA Expression in CLL Cells

**Figure 3:**
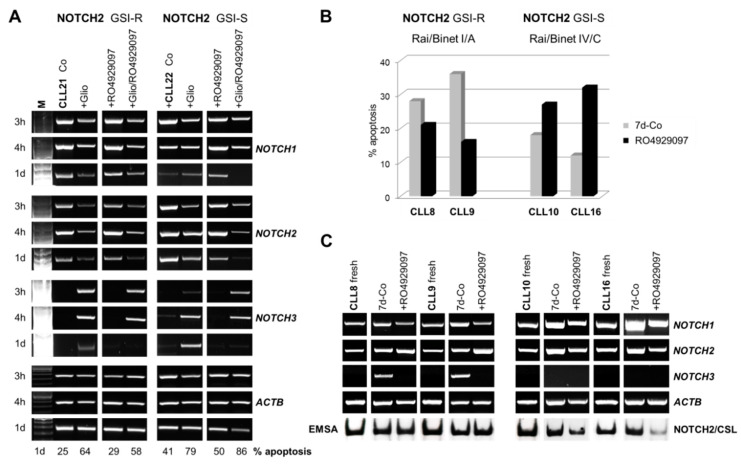
RO4929097 inhibited gliotoxin-induced and basal *NOTCH3* mRNA expression in CLL cells. (**A**) Time kinetic RT-PCR analysis showing *NOTCH1*, *NOTCH2*, and *NOTCH3* mRNA expression in response to 0.2 µM gliotoxin and/or 0.5 µM RO4929097 treatment in relation to the GSI sensitivity of nuclear NOTCH2. For co-treatment, CLL cells were first incubated with RO4929097 immediately before adding gliotoxin. The effect of the compounds on the percentage of apoptotic CLL cells after 1 day in culture is indicated. (**B**) FACS analysis indicating the inhibition/induction of apoptosis by RO4929097 (0.5 µM) in fresh CLL samples derived from two representative NOTCH2 GSI-R Rai/Binet I/A and two representative NOTCH2 GSI-S Rai/Binet IV/C CLL patient samples after 7 days in culture. (**C**) Corresponding RT-PCR showing the effect of RO4929097 (0.5 µM) on *NOTCH1*, *NOTCH2*, and recovered *NOTCH3* mRNA expression on Day 7. The GSI resistance/sensitivity of nuclear NOTCH2/CSL DNA-complexes is indicated by EMSA. *ACTB* was included as the internal control.

We next confirmed in a time kinetic RT-PCR experiment in two additional representative CLL samples (CLL21, 22) our previous observation that gliotoxin inhibited *NOTCH2*, to a lesser extent *NOTCH1*, and induced *NOTCH3* mRNA expression within 4 h of incubation (Figure 3A) [32]. Interestingly, RO4929097 counteracted gliotoxin-induced *NOTCH3* transcription after one day (Figure 3A). This suggests that RO4929097 interrupted a positive feedback loop of *NOTCH3* mRNA expression. NOTCH receptors regulate context and cell type specific their own expression and each other in positive and negative feedback loops [19]. Moreover, RO4929097 decreased (58 versus 64%) or increased (86 versus 79%) the effect of gliotoxin on the percentage of apoptotic cells, depending on the GSI sensitivity of nuclear NOTCH2 (Figure 3A). Therefore, we asked whether NOTCH3 might also account for the GSI-mediated inhibition of spontaneous apoptosis in early clinical stage-derived CLL long-term suspension cultures.

*NOTCH3* mRNA was not detectable in frozen samples from freshly isolated CLL cells of our initial drug screening cohort (Figure 3C). After 7 days, however, the anti-apoptotic effect of RO4929097 in two representative Rai/Binet I/A patient samples expressing GSI-R nuclear NOTCH2 (CLL8 and CLL9, Figure 3B) was clearly associated with inhibition of spontaneously recovered *NOTCH3* mRNA expression, together with unchanged or even enhanced *NOTCH2* mRNA and NOTCH2/CSL DNA-complex expression (Figure 3C, left panel).

In contrast, the induction of apoptosis by RO4929097 in two representative Rai/Binet IV/C patient samples expressing GSI-S nuclear NOTCH2 (CLL10 and CLL16, Figure 3B) was associated with a decrease in *NOTCH2* mRNA and NOTCH2/CSL DNA-complex expression without any detectable *NOTCH3* gene activity after 7 days in culture (Figure 3C, right panel). In accordance with published data [9,16], the *NOTCH1* mRNA was GSI sensitive in all cases and was more expressed in the Rai/Binet IV/C-derived CLL cells (Figure 3C).

Together, this combined approach suggests that recovery of *NOTCH3* mRNA expression is involved in the inhibition of spontaneous apoptosis by GSI in CLL long-term suspension cultures and may be associated with GSI resistance of NOTCH2 and early stage derived CLL samples.

### 3.4. Induction of Surface NOTCH3 Expression by Gliotoxin is Associated with Downregulation of CD23 and Increased Apoptosis of CLL Cells

**Figure 4:**
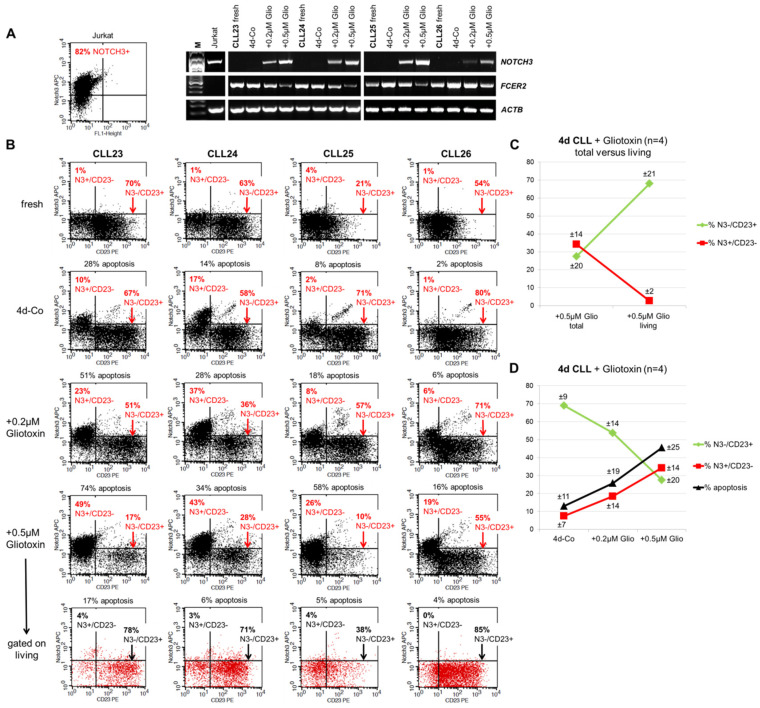
NOTCH3 and CD23 surface expression are mutually exclusive on CLL cells. (**A**) RT-PCR and (**B**) FACS showing NOTCH3 and *FCER2* (CD23) expression in freshly isolated CLL cells and after 4 days in culture in relation to surface CD23 expression and spontaneous as well as gliotoxin-induced apoptosis. The T-ALL cell line Jurkat served as positive control for *NOTCH3* mRNA and NOTCH3 surface expression. (**C**) Gating on the remaining living cells after gliotoxin treatment according to their forward/side scatter properties revealed that living CLL cells were enriched for NOTCH3-/CD23+ cells. (**D**) Summary of the FACS data, demonstrating a direct correlation of the percentage of NOTCH3+/CD23- and apoptotic CLL cells and an indirect correlation of these two parameters with the percentage of NOTCH3-/CD23+ CLL cells. Data presented as means (±SD).

We next analyzed NOTCH3 and *FCER2* (CD23) expression in CLL cells (n = 4) in relation to spontaneous as well as gliotoxin induced apoptosis on the mRNA and protein level by RT-PCR and FACS (Figure 4; see Supplemental Figure S1 for a detailed FACS analysis of CLL24 cells). NOTCH3 was almost undetectable on the mRNA (Figure 4A) and on the cell surface protein level (Figure 4B) in freshly isolated CLL cells. After 4 days in culture, we found an increase of surface NOTCH3 expression together with a decrease in surface CD23 on CLL23 and CLL24 cells (Figure 4B). On the mRNA level, *NOTCH3* expression was below the detection limit in the 4 days control. The loss of CD23 expression seemed to be a prerequisite for NOTCH3 expression since NOTCH3 was primarily detected on CD23-negative CLL cells.

In contrast, we detected a low expression of CD23 on freshly isolated cells from Duvelisib (CLL25) or Ibrutinib (CLL26) treated CLL patients (Figure 4B). However, *FCER2* (CD23) expression spontaneously recovered on the mRNA and protein level in these 2 samples after 4 days in culture which might be attributed to the loss of the inhibitory effect of these drugs on CD23 over time (Figure 4A,B).

Gliotoxin induced the *NOTCH3* gene (Figure 4A), enhanced NOTCH3 surface expression, and upregulated apoptosis in a dose-dependent manner in all cases (Figure 4B). The remaining living CLL cells were enriched for CD23-positive and NOTCH3-negative cells (Figure 4B,C and Figure S1), confirming that CD23 expression is associated with CLL cell viability whereas NOTCH3 expression is associated with CLL cell apoptosis.

In summary, we found a direct correlation between the percentage of surface NOTCH3-positive and apoptotic CLL cells and an indirect correlation of these two parameters with the percentage of CD23-positive and living CLL lymphocytes (Figure 4D).

### 3.5. Targeting NOTCH3 Signaling Decreased NR4A1 mRNA Expression and Counteracted Gliotoxin Induced Apoptosis in CLL Cells

**Figure 5:**
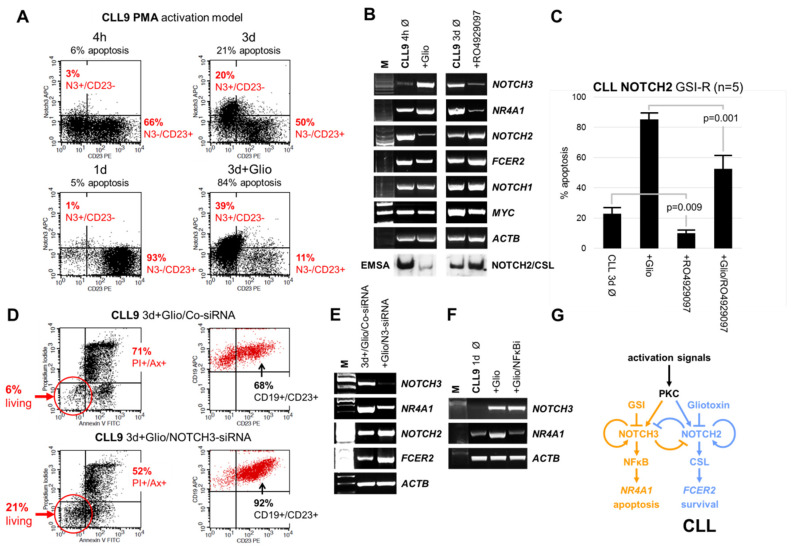
NOTCH3 inhibition counteracts apoptosis in PMA-activated CLL cells. (**A**) FACS analysis indicating the time-dependent effect of PMA (1 ng/mL) on surface NOTCH3 and CD23 expression in CLL9 cells. (**B**) Corresponding RT-PCR showing the opposite effects of gliotoxin (0.2 µM) and RO4929097 (0.5 µM) on the *NOTCH2/FCER2 (CD23)* axis and the *NOTCH3/NR4A1* axis in PMA-activated CLL9 cells. The mRNA expression of the CLL proliferation center marker *MYC* is indicated [41,42]. (**C**) FACS analysis demonstrating the inhibition of spontaneous/gliotoxin induced apoptosis by RO4929097 in NOTCH2 GSI-R CLL cells. (**D**) *NOTCH3*-siRNA counteracted gliotoxin induced apoptosis in PMA-activated CLL9 cells and enhanced surface CD23 expression on the remaining living cells. (**E**) Corresponding RT-PCR showing the opposite effects of *NOTCH3* gene silencing on the *NOTCH3/NR4A1* axis and on the *NOTCH2/FCER2* axis. (**F**) RT-PCR showing the inhibition of gliotoxin induced *NR4A1* mRNA expression by 0.1µM NFκB activation inhibitor (NFκBi). (**G**) Hypothetical model summarizing the proposed counteracting roles of NOTCH2 and NOTCH3 in CLL cells. Non-canonical NOTCH3 signaling which involves NFκB dependent *NR4A1* expression is shown in yellow color. Canonical NOTCH2 signaling which involves CSL dependent *FCER2* (CD23) expression is marked in blue. The NOTCH2 dominance in CLL is indicated. Positive and negative feedback loops of NOTCH receptor expression and function are indicated with circular arrows and bars.

We hypothesized that NOTCH2 and NOTCH3 have opposite roles in the binary cell fate decision between positive and negative selection of the activated CLL cells. Therefore, we investigated the effects of targeting NOTCH3 by RO4929097, or more specifically, by siRNA in PMA-stimulated CLL cells [32]. In this model, CLL cells form tight clusters and express the CLL proliferation center marker *MYC* (Figure 5B), resembling the situation found in lymphoid tissues [41,42]. To avoid background effects on NOTCH2 signaling, we selected five CLL cases expressing GSI-R NOTCH2 (CLL7, 8, 9, 13, 21).

A time kinetic confirmed that CD23 was expressed on almost all representative CLL9 cells after one day of PMA stimulation (Figure 5A) [18]. Interestingly, prolonged stimulation with PMA for 3 days led to the downregulation of CD23, upregulation of NOTCH3, and an increase in the percentage of apoptotic CLL9 cells. This effect was clearly enhanced by gliotoxin treatment (Figure 5A).

As expected, gliotoxin induced the *NOTCH3/NR4A1* axis, downregulated the *NOTCH2/FCER2* (CD23) axis, and inhibited the NOTCH2/CSL transcription factor complex within 4 h of incubation (Figure 5B, left panel) [32]. In accordance with the FACS data, the *NOTCH3/NR4A1* axis was also upregulated in PMA-stimulated CLL9 cells after 3 days in culture without gliotoxin treatment (Figure 5B, right panel). Neither gliotoxin nor PMA induced DNA-bound NOTCH3/CSL complexes in EMSA. RO4929097 inhibited the *NOTCH3/NR4A1* axis, upregulated the *NOTCH2/FCER2* (CD23) axis, and enhanced the NOTCH2/CSL transcription factor complex (Figure 5B, right panel), which stands in sharp contrast to the effect of gliotoxin. The NOTCH1 target gene *MYC* was downregulated by RO4929097 (Figure 5B, right panel) [9,43,44,45].

The opposite effects of gliotoxin and RO4929097 on the *NOTCH2/FCER2* (CD23) axis and the *NOTCH3/NR4A1* axis were reflected by the opposite effects of these compounds on CLL cell viability. As shown in Figure 5C, gliotoxin remarkably induced apoptosis (*n* = 5, mean% ± SD: 85 ± 5% versus 23 ± 6%), while RO4929097 significantly decreased spontaneous (10 ± 2% versus 23 ± 6%; *p* = 0.009) as well as gliotoxin-induced apoptosis (53 ± 9% versus 85 ± 5%; *p* = 0.001) in CLL cells.

*NOTCH3* gene silencing by siRNA decreased the apoptotic effect of the gliotoxin, leading to a 3.5-fold increase in (Ax-/PI-) living CLL9 cells with increased surface CD23 expression after 3 days in culture (Figure 5D). Corresponding RT-PCR analysis confirmed that *NOTCH3* gene silencing downregulated the *NOTCH3/NR4A1* axis and upregulated the *NOTCH2/FCER2* (CD23) axis (Figure 5E), resembling the effect of GSI treatment (Figure 5B, right panel). Interestingly, gliotoxin-mediated upregulation of the *NR4A1* gene was completely blocked by an NFκB activation inhibitor (Figure 5F), suggesting that NOTCH3 regulates *NR4A1* transcription via non-canonical NOTCH3/NFκB signaling [23,46,47]. This would explain the lack of DNA-bound NOTCH3/CSL complexes in EMSA. All other control experiments show similar trends.

Collectively, these data strongly suggest that inhibition of the anti-apoptotic canonical NOTCH2/CSL signaling (*NOTCH2/FCER2* axis) by gliotoxin recovers a GSI sensitive pro-apoptotic non-canonical NOTCH3 function which may involve NFκB dependent *NR4A1* expression (*NOTCH3/NR4A1* axis) in CLL cells. A hypothetical model summarizing the proposed counteracting roles of NOTCH2 and NOTCH3 in CLL cells is given in Figure 5G.

### 3.6. Gliotoxin Modulates Chromatin Accessibility at Gene Regulatory Elements Containing Potential NOTCH/CSL and NR4A1 Binding Sites

**Figure 6:**
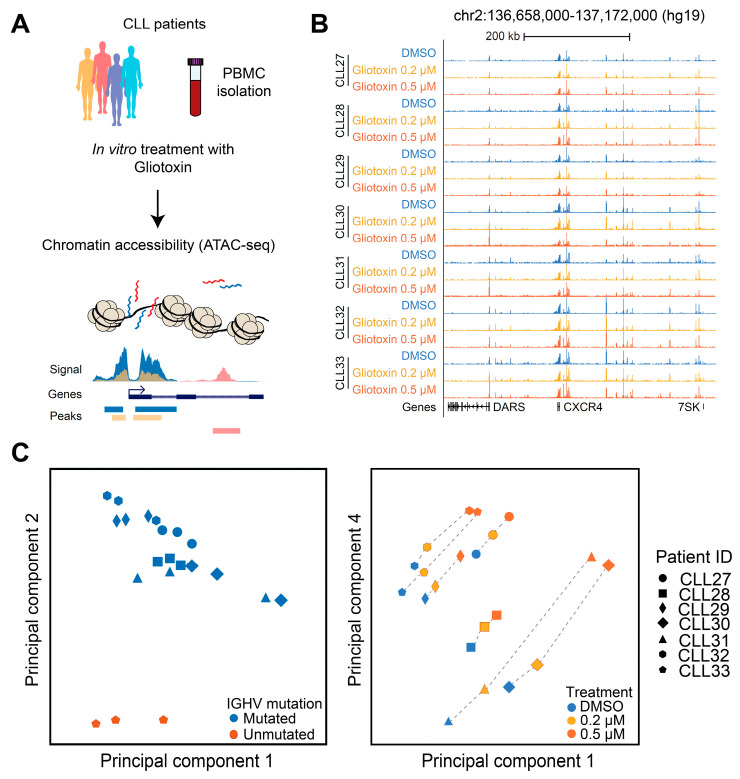
Chromatin accessibility changes in primary CLL cells upon in vitro treatment with gliotoxin. (**A**) ATAC-seq workflow to study the chromatin accessibility changes of CLL cells in response to gliotoxin treatment. (**B**) Representative genome browser visualization of the ATAC-seq signals in patient-derived CLL samples (*n* = 7) treated with DMSO and two different concentrations of gliotoxin. A genomic region spanning ~50 kb around the *CXCR4* locus is shown. (**C**) Unsupervised principal component analysis based on the chromatin accessibility for all 21 samples at all accessible sites in all samples. Samples are color coded according to their IGHV mutation status (left panel), or according to the in vitro treatment condition (right panel).

We have recently shown that genome-wide mapping of gene-regulatory elements using the transposase-accessible chromatin (ATAC-seq) assay is a useful tool to investigate gene regulation in CLL cells [35]. Therefore, we analyzed gliotoxin-induced chromatin changes in CLL cells after 3 days of incubation (*n* = 7, Figure 6A), where we identified 62,760 unique chromatin accessible regions (Figure S2A). These sites represent mainly enhancers and promoters (Figure S2B, a representative locus spanning the *CXCR4* gene region is shown in Figure 6B). Unsupervised principal component analysis (PCA) confirmed that *IGHV* mutation status is the major source of heterogeneity in chromatin accessibility in CLL cells, as described previously (Figure 6C, left panel) [35]. However, Principle Components 1 and 4 clearly revealed dose-dependent changes in chromatin accessibility in response to gliotoxin treatment (Figure 6C, right panel).

**Figure 7:**
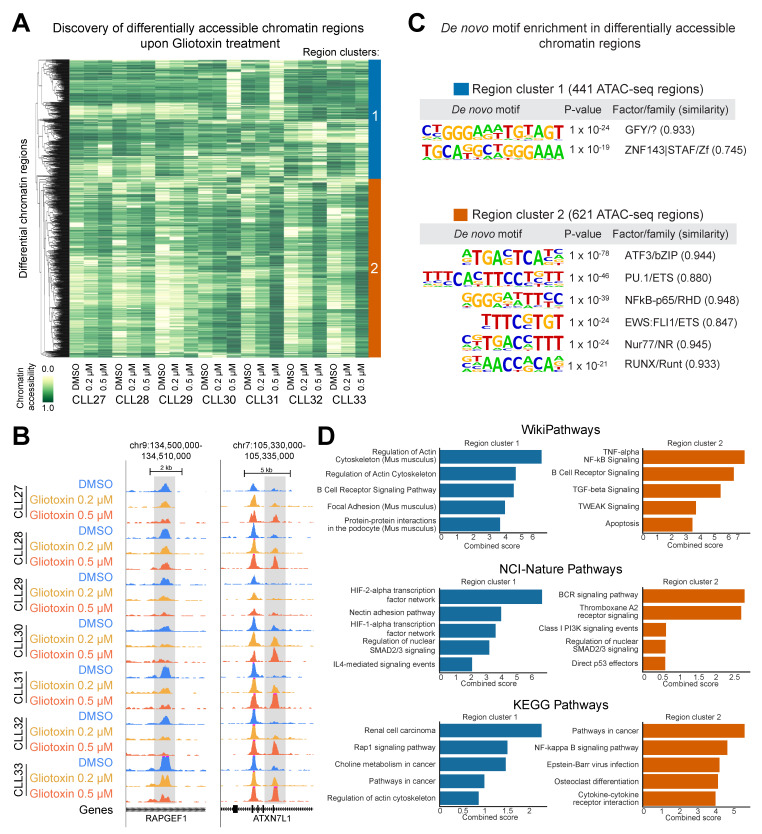
Chromatin gliotoxin treatment-related changes in chromatin accessibility in CLL cells. (**A**) Clustered heatmap based on all the differentially accessible regions between treatment conditions. (**B**) Representative browser visualization of genomic regions that lost (left panel for *RAPGEF1*) or gained (right panel for *ATXN7L1*) chromatin accessibility upon gliotoxin treatment. (**C**) De novo motif enrichment analysis of differentially accessible regions from Region Clusters 1 and 2. (**D**) Most highly enriched pathways for genes associated with Region Clusters 1 and 2.

Clustering of significantly changing regions (Figure 7A) segregated the sites into those that lose accessibility (Region Cluster 1, marked in blue) and those that gain accessibility (Region Cluster 2, marked in orange) in response to gliotoxin treatment (representative genomic loci of each cluster are shown in Figure 7B). By overlapping the differential ATAC-seq sites with publicly available transcription factor binding experiments [38], we observed that Region Cluster 1 largely overlaps with the functional NOTCH/CSL binding sites in T-cell acute leukemia cells (Figure S3A). On the other hand, Region Cluster 2 largely overlaps with the functional NFκB binding sites in lymphoblastoid cell lines (Figure S3B).

To get unbiased insights into the potential regulators of differentially accessible regions we carried out de novo motif analysis to discover the enriched DNA binding elements. Differential accessible gene-regulatory elements in Region Cluster 1 were found to be enriched for the promoter-associated motif GFY (general factor Y) and the recognition site for ZNF143, both of which contain the NOTCH/CSL consensus binding site TGGGAA [48]. In Region Cluster 2, we found an enrichment of consensus sites for the transcription factors ATF3, PU.1, NFκB, FLI1, NR4A1 (nur77), and RUNX (Figure 7C).

By linking the differentially accessible regions to their nearest gene, we found that Region Cluster 1 maps toward the genes involved in the regulation of the actin cytoskeleton (*ITGB1*, *ACTN4*, *MSN*, *SSH1*, and others), HIF-1/2-alpha signaling (*VEGFA*, *HMOX1*, *TRFC*, *PLCG2*, and others) and RAP1 signaling (*RAPGEF1*, *RAP1A*, *VAV2*, and others) (Figure 7D, see Table S1). Region Cluster 2 was enriched for genes involved in B-cell activation/differentiation (*LYN*, *IRF4*, *BCL6*, *ETV6*, *ARID3A*, *POU2F2*, *THEMIS2*, *IL21R*, and others), NFκB signaling (*TRAF1-3*, *RELB*, *CARD11*, and others), TGFß signaling (*TGFB1*, *SMAD3*, *PML*, and others), and apoptosis (*FASL*, *TP73*, *BBC3*, *BID*, *MIR34A*, *TNFRSF8*, *GZMB*, and others) (Figure 7D, see Table S2).

## 4. Discussion

In the last decade, much effort has been spent into the development of GSIs as tools for therapy for NOTCH-associated human neoplasias [29,30,31]. However, four NOTCH family members are present in mammalian cells, where the individual receptors may have opposite functions concerning their role as oncogenes or tumor suppressors in a context- and microenvironment-dependent manner [26,30,31]. Moreover, transformed cells may express truncated NOTCH forms that do not depend on γ-secretase for processing and function [49,50]. A search for an alternative to GSI revealed that the *Aspergillum*-derived secondary metabolite gliotoxin is a potent inhibitor of DNA-bound NOTCH2/CSL transcription factor complexes, and efficiently induced apoptosis in CLL lymphocytes and in NOTCH2-associated solid tumor cell lines—independent of their sensitivity to GSIs [32,51]

In this study, we show that gliotoxin as a nuclear NOTCH2 inhibitor may have an additional therapeutic advantage over GSI as pan-NOTCH inhibitors in CLL. We were able to demonstrate that the GSI RO4929097 targets an unexpected non-canonical tumor-suppressing NOTCH3 activity, which is involved in spontaneous as well as gliotoxin-induced apoptosis in CLL cells.

On the CLL cell surface, upregulation of NOTCH3 was associated with downregulation of CD23, suggesting that downregulation of the *NOTCH2/FCER2* (CD23) axis, either spontaneously due to the lack of appropriate activation stimuli in vitro [18], or by gliotoxin treatment [32], is a prerequisite for NOTCH3 expression in CLL cells. The strong association between surface NOTCH3 expression and apoptosis suggests that NOTCH3 signaling is involved in the execution phase of CLL cell apoptosis by prolonging the expression of the newly identified NOTCH3 target gene *NR4A1*, a multi-functional tumor-suppressor gene implicated in the regulation of B-cell tolerance to self-antigens [52,53,54].

ATAC-seq confirmed that gliotoxin targets canonical NOTCH signaling as indicated by reduced accessibility at potential NOTCH/CSL consensus sites (TGGGAA) [48]. This includes the promoter-associated motif GFY, and the consensus site for ZNF143, a transcription factor known to modulate NOTCH target gene expression in competition with CSL [55]. In contrast, gliotoxin increased the chromatin accessibility at the potential ATF3, PU.1, NFκB, FLI1, RUNX, and NR4A1 binding sites in the vicinity of genes involved in B-cell activation, differentiation, and apoptosis. This might reflect, at least in part, the loss of a NOTCH2-mediated differentiation arrest and the induction of non-canonical NOTCH3/NFκB signaling [23]. It has been shown that NOTCH3 activates NFκB, a positive regulator of the *NR4A1* gene [46], through an IKKα-dependent alternative pathway [47]. This would not only explain the gain in chromatin accessibility at NFκB and NR4A1 consensus sites and the lack of DNA-bound NOTCH3/CSL complexes in response to gliotoxin treatment, but also the NOTCH3-dependent *NR4A1* gene activity in CLL cells. In line with this assumption, we found that inhibition of NFκB counteracted gliotoxin-mediated upregulation of *NR4A1* mRNA in CLL lymphocytes. However, this important issue needs further mechanistic exploration.

NOTCH2 and NOTCH3 signaling antagonize each other in different cell systems [56,57,58,59], suggesting that these NOTCH receptors also have opposite functions in the antigen-dependent regulation of CD5+ (B-1a) B-cell homeostasis. Under physiological conditions, NOTCH2 signaling might be induced by ligand-expressing surrounding cells in order to protect the proliferative/regenerative reservoir of CD5+ B-cells from NR4A1-mediated activation-induced cell death (AICD) [32,52,60]. This scenario might take place in the marginal zone (MZ) of the spleen [61], where DLL1-expressing bystander cells have been identified [62]. In contrast, apoptotic NOTCH3 signaling might counteract the uncontrolled expansion of CD5+ B-cells in the periphery. In line with this hypothesis, we found that NOTCH3 inhibition by RO4929097, or more specifically, by siRNA, downregulated the *NOTCH3/NR4A1* axis, enhanced the *NOTCH2/FCER2* (CD23) axis, and counteracted apoptosis in CLL cells. The *NOTCH3* gene is frequently epigenetically silenced in B-cell acute lymphocytic leukemia (B-ALL) cells, pointing to a broader tumor-suppressor role of NOTCH3 in B-cells [63]. Moreover, the CLL downregulated/deleted MicroRNA-16 [64] has been shown to exert its pro-apoptotic function by NOTCH2 inhibition in pre-eclampsia, where an inverse correlation between NOTCH2 and NOTCH3 expression also has been found [59].

In terms of CLL biology, constitutive active NOTCH2 might dominantly suppress apoptotic NOTCH3 signaling, thereby enabling the (self-) antigen-driven progredient expansion of the malignant clone. The affinity and avidity of the corresponding (self-) antigens might be the basis for the NOTCH2 dominance seen in CLL cells. A short time exposure to B-cell activation mimetic PMA favors the *NOTCH2/FCER2* (CD23) axis, whereas long-term stimulation with PMA shifts the *NOTCH2/FCER2* (CD23) axis to the *NOTCH3/NR4A1* axis in CLL cells. One likely mediator of this bi-phasic PMA effect might be the activation and subsequent downregulation of the B-cell receptor-associated protein kinase C-delta (PKC-δ) [65,66,67], a positive regulator of nuclear NOTCH2 activity [10,18,27,68]. Therefore, compounds that interfere with B-cell activation might affect the expression of both NOTCH receptors in CLL cells. This would explain why CLL cells pre-treated with Duvelisib (a dual PI3K-δ/γ inhibitor) [69] or Ibrutinib (a Bruton’s tyrosine kinase inhibitor) [2,44] express lower amounts of CD23 and NOTCH3 and are less sensitive to apoptosis.

The additional effect of NOTCH1 signaling in this scenario is less clear. NOTCH1 is not detectable in nuclear NOTCH/CSL transcription factor complexes in CLL cells [7,8,18,32]. However, *NOTCH1* is frequently mutated and/or overexpressed in advanced stage CLL cells, where it has a CLL-driving role by regulating *MYC* expression [8,9,16,17,43,44,45]. In this context, NOTCH1 may indirectly account for the relative GSI sensitivity of NOTCH2, keeping in mind that active NOTCH1 is a positive regulator of the *NOTCH2* gene in CLL cells (Figure 5G) [12]. 

## 5. Conclusions

In summary, we show that the nuclear NOTCH2 inhibitor gliotoxin has global effects on the NOTCH signaling network in CLL cells, including the recovery of a newly identified non-canonical tumor suppressing NOTCH3 activity. This proof-of-concept may be the basis for the design of innovative therapies aimed at specifically targeting oncogenic NOTCH signaling in CLL cells.

## Figures and Tables

**Table 1:** Clinical and prognostic parameters of the chronic lymphocytic leukemia (CLL) samples enrolled in this study.

Pat. ID	Age	Gender	Rai/Binet Stage	*IGHV* Status	Cytogenetic Alterations	*NOTCH1* Mutations	NOTCH2 GSI-R/S*	Treatment
CLL1	76	male	II/B	U, *VH2-5*	normal	*N1ΔCT*	GSI-S	no
CLL2	69	male	II/B	U, *VH1-69*	normal	*N1ΔCT*	GSI-R	no
CLL3	70	male	IV/C	M, *VH4-34*	Tri12	wt	ND	no
CLL4	64	male	II/B	M, *VH3-23*	13q-, 17p-	wt	ND	no
CLL5	51	female	IV/C	U, *VH1-69*	normal	wt	ND	no
CLL6	56	male	IV/C	M, *VH3-21*	13q-, 11q-	wt	GSI-S	no
CLL7	68	male	II/B	M, *VH3-48*	13q-, 11q-	wt	GSI-R	no
CLL8	84	male	I/A	NA	normal	wt	GSI-R	no
CLL9	81	female	I/A	M, *VH3-15*	13q-	wt	GSI-R	no
CLL10	73	female	IV/C	M, *VH3-23*	normal	wt	GSI-S	no
CLL11	66	female	I/A	M, *VH3-48*	13q-	wt	GSI-R	no
CLL12	70	male	I/A	M, *VH1-8*	13q-	wt	GSI-R	no
CLL13	66	female	II/B	M, *VH3-23*	13q-	wt	GSI-R	no
CLL14	75	female	IV/C	NA	13q-	wt	GSI-S	no
CLL15	65	male	I/A	U, *VH1-69*	14q32-	wt	GSI-R	no
CLL16	52	male	IV/C	U, *VH1-69*	normal	wt	GSI-S	no
CLL17	55	male	II/B	U, *VH3-11*	13q-, 11q-	wt	GSI-R	Ibrutinib
CLL18	40	female	I/A	U, *VH3-20*	normal	wt	GSI-S	no
CLL19	68	female	III/B	U, *VH1-2*	13q-	*N1ΔCT*	GSI-S	no
CLL20	60	male	IV/C	U, *VH1-46*	13q-, 11q-	*N1ΔCT*	GSI-S	no
CLL21	52	male	II/B	NA	13q-	ND	GSI-R	no
CLL22	70	female	II/B	M, *VH3-13*	13q-	ND	GSI-S	no
CLL23	54	male	I/A	U, *VH3-53*	normal	ND	ND	no
CLL24	77	female	IV/C	NA	17p-	ND	ND	no
CLL25	54	male	I/A	U, *VH4-39*	11q-	ND	ND	Duvelisib
CLL26	69	male	II/B	U, *VH3-21*	13q-	ND	ND	Ibrutinib
CLL27	70	male	II/B	M, *VH3-13*	13q-	ND	ND	no
CLL28	61	female	I/A	M, *VH3-7*	normal	ND	ND	no
CLL29	77	female	II/B	M, *VH3-74*	normal	ND	ND	no
CLL30	87	female	IV/C	M, *VH3-11*	13q-	ND	ND	Idealisib
CLL31	68	female	II/B	M, *VH3-48*	13q-	ND	ND	no
CLL32	83	female	II/B	M, *VH4-59*	13q-	ND	ND	Ibrutinib
CLL33	60	male	II/B	U, *VH1-69*	13q-/11q-	ND	ND	Idealisib

33 CLL patients were matched in terms of age, gender, Rai/Binet stages, IgVH mutational status and cytogenetic aberrations. CLL1-20 were used for initial drug screening and CLL21-33 were additionally used for follow-up and validation experiments. Abbreviations: U, *IGHV* unmutated; M, *IGHV* mutated; ND, not determined; NA, not amplifiable; *N1ΔCT* indicates the recurrent *NOTCH1* microdeletion; wt indicates wild type. NOTCH2 GSI-R/S* indicates the expression of the GSI-resistant/sensitive DNA-bound NOTCH2/CSL complexes.

## Supplementary Materials

The following are available online at https://www.mdpi.com/2073-4409/9/6/1484/s1, Figure S1: Detailed FACS data of CLL24 cells, Figure S2: Genomic distribution and characteristics of open chromatin sites in CLL cells in response to gliotoxin treatment, Figure S3: Overlap of differential ATAC-seq sites with public transcription factor binding datasets in gliotoxin treated CLL cells, Table S1: ATAC-seq gliotoxin response region cluster 1, Table S2: ATAC-seq gliotoxin response region cluster 2.
