# Supplementary material for: Targeting Nuclear NOTCH2 by Gliotoxin Recovers a Tumor-Suppressor NOTCH3 Activity in CLL
Source: Cells. 2020 Jun 18;9(6):1484. doi: 10.3390/cells9061484 (PMC7348714; doi:10.3390/cells9061484)
Supplement: Supplementary file 1 [file cells-09-01484-s001.zip › Fig S1 Hubmann et al CELLS2020.pdf]

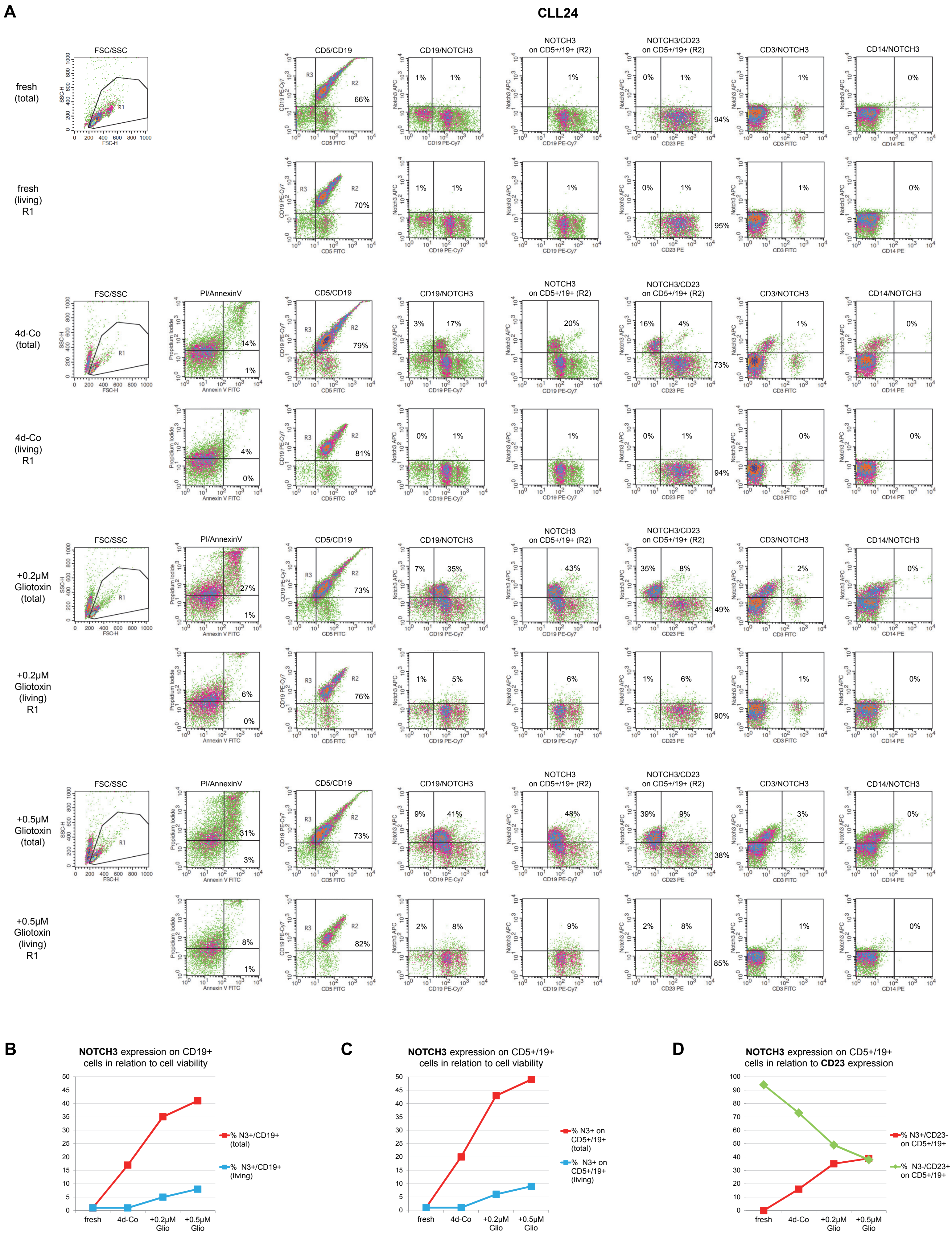

Supplemental Figure 1. (A) Detailed FACS data of CLL24 showing the percentage of NOTCH3+ cells on CD19+ (B-cells), CD5+/19+ (CLL cells), CD3+ (T-cells), and CD14+ (Monocytes/Macrophages) lymphocytes in relation to cell viability on freshly isolated PBMC's and after four days in suspension culture ± gliotoxin. FACS dot blots are indicated as coloured cell density blots. A summary of the percentage of NOTCH3 on (B) CD19+ and (C) CD5+/19+ cells in relation to cell viability is indicated. (D) NOTCH3 indirectly correlates with CD23 expression on CD5+/19+ CLL cells.
