# Supplementary material for: Targeting Nuclear NOTCH2 by Gliotoxin Recovers a Tumor-Suppressor NOTCH3 Activity in CLL
Source: Cells. 2020 Jun 18;9(6):1484. doi: 10.3390/cells9061484 (PMC7348714; doi:10.3390/cells9061484)
Supplement: Supplementary file 1 [file cells-09-01484-s001.zip › Fig S2 Hubmann et al CELLS2020.pdf]

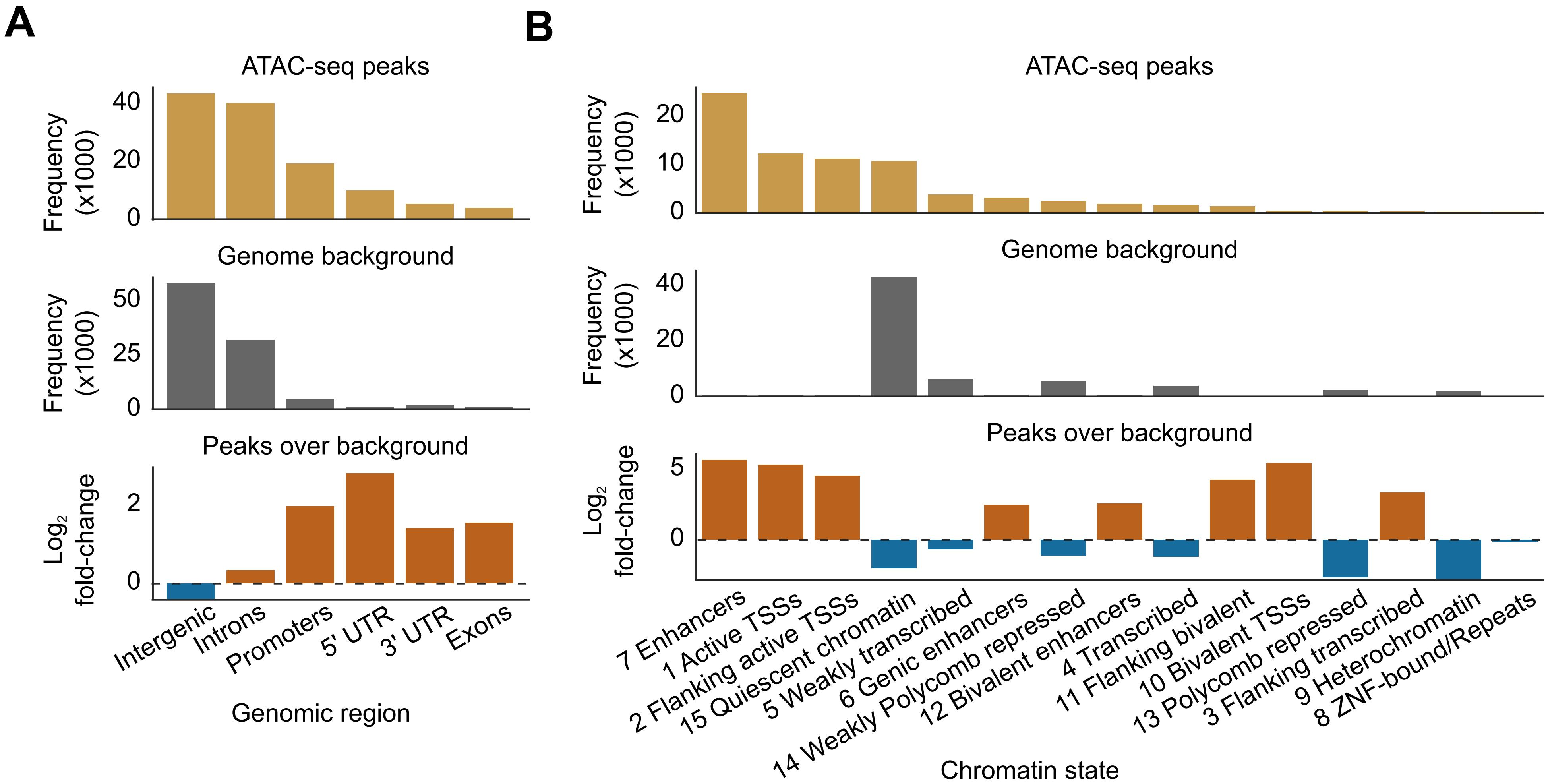

Supplemental Figure 2. Genomic distribution and characteristics of open chromatin sites in CLL cells in response to gliotoxin treatment. (**A**) Absolute (frequency) and relative (log2 fold change) co-localization of unique chromatin-accessible regions in CLL with gene annotations. (**B**) Absolute (frequency) and relative (log2 fold change) co-localization of unique chromatin-accessible regions in CLL with chromatin state segmentations for CD19+ B cells from the Roadmap Epigenomics project.
