# Supplementary material for: Targeting Nuclear NOTCH2 by Gliotoxin Recovers a Tumor-Suppressor NOTCH3 Activity in CLL
Source: Cells. 2020 Jun 18;9(6):1484. doi: 10.3390/cells9061484 (PMC7348714; doi:10.3390/cells9061484)
Supplement: Supplementary file 1 [file cells-09-01484-s001.zip › Fig S3 Hubmann et al CELLS2020.pdf]

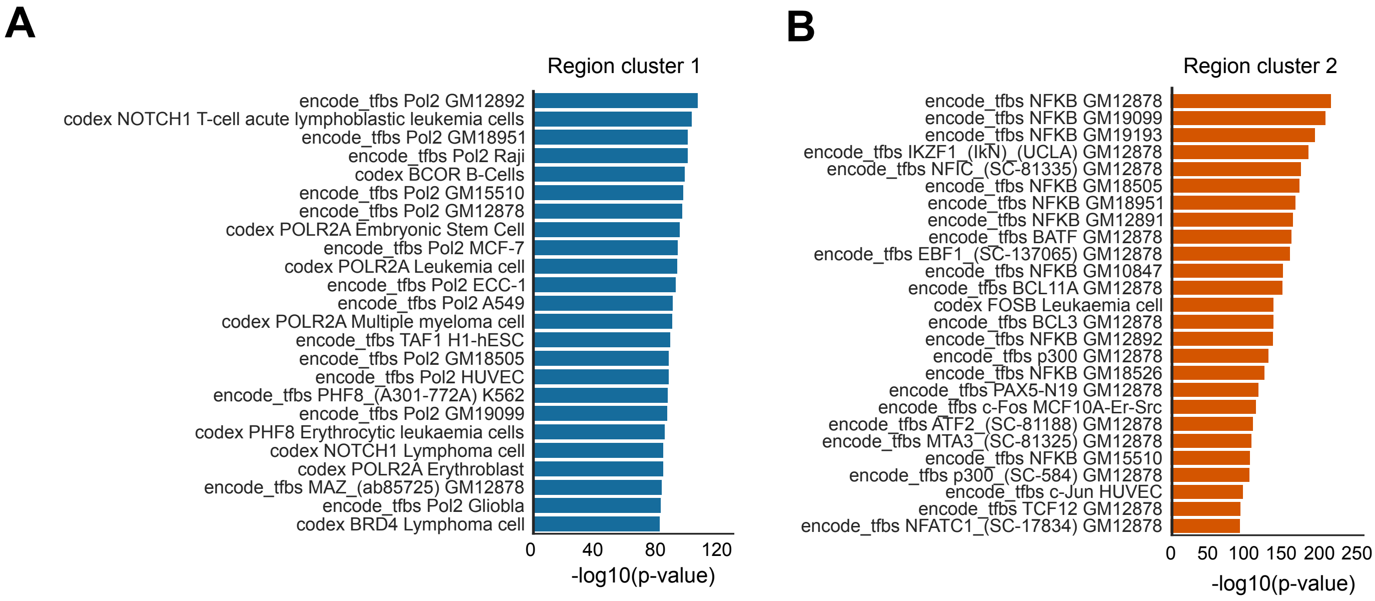

Supplemental Figure 3. Overlap of differential ATAC-seq sites with public transcription factor binding datasets in gliotoxin treated CLL cells. Most highly enriched region sets (CODEX and encode transcription factor binding) of (**A**) region cluster 1 and (**B**) region cluster 2.
